# Echocardiographic Features of Longevity: A Cross-Sectional Study of Centenarians

**DOI:** 10.7759/cureus.30842

**Published:** 2022-10-29

**Authors:** Jorge Perez, Benjamin Hurwitz, Douglas Salguero, Marissa Donattele, Esteban Escolar, Rafle Fernandez, Christos G Mihos

**Affiliations:** 1 Division of Cardiology, Mount Sinai Heart Institute, Columbia University College of Physicians and Surgeons, Miami Beach, USA; 2 Department of Internal Medicine, Mount Sinai Medical Center, Miami Beach, USA

**Keywords:** valvular heart disease, left ventricular remodeling, echocardiography, diastology, centenarians, cardiac geometry

## Abstract

Background: Centenarians represent an under-studied population within cardiovascular medicine. This study aimed to describe the echocardiographic characteristics of a cohort of centenarians at a tertiary referral center.

Materials and methods: The institutional Echocardiography database was retrospectively reviewed and identified 100 consecutive centenarians referred for transthoracic echocardiography between January 2009 and December 2020. Cardiac chamber quantification, diastology, and valvular heart disease were assessed according to the American Society of Echocardiography guidelines. Independent t-tests and Mann-Whitney U-tests compared data between males and females.

Results: The mean age was 101.5 ± 1.7 years, 78% were female, and the most common co-morbidities were hypertension (77%), coronary artery disease (46%), and congestive heart failure (42%). The mean left ventricular (LV) ejection fraction measured 56.9 ± 11.3% (females vs males, 58.4 ± 9.8 vs 51.6 ± 14.6%, p = 0.01). Males had larger LV end-diastolic (2.8 ± 0.6 vs 2.5 ± 0.5, p = 0.03) and end-systolic diameter (1.9 ± 0.6 vs 1.6 ± 0.4, p = 0.001) indices; a smaller relative wall thickness (0.54 ± 0.18 vs 0.69 ± 0.36, p = 0.06); and a lower E/e’ ratio (13.3 [10.3-19.6] vs 17.3 [13.2-23], p = 0.05), when compared with females. The prevalence of severe valvular lesions was 13.5%, and similar between genders. However, in patients with aortic stenosis, the transaortic pressure gradients were significantly higher in females (mean gradient: 32.0 ± 17.7 vs 18.7 ± 9.2 mmHg, p = 0.04).

Conclusions: The present study on centenarians affords a cross-sectional evaluation of cardiac structure and function in a growing population, and highlights important differences between male and female patients.

## Introduction

In our progressively aging population, it is common to encounter a centenarian in clinical practice. As modern medicine advances and therapies aimed at risk factor modification become widespread, the elderly population is expected to continue to grow at a rapid pace. Indeed, the number of centenarians has doubled every decade since the 1950s and is estimated to increase five-fold by the year 2050 [1-3]. Among this population, cardiovascular disease is frequent and remains the most common cause of mortality [4,5]. However, it remains unclear how expected age-related changes, such as arterial stiffening, ventricular remodeling, and valvular degeneration, manifest in the centenarian heart [6].

Transthoracic echocardiography is universally utilized as a first-line imaging assessment in patients suspected of cardiovascular pathology, carries minimal inherent risk, and provides an unmatched wealth of information regarding cardiac remodeling, hemodynamics, and valvular heart disease. This imparts a significant benefit in the diagnosis and treatment of cardiovascular disorders across age groups. Nevertheless, cardiac structural and physiologic changes have not been studied in centenarians to the extent that they have in younger populations, and published data have generally been limited by small sample sizes and differing methodologies [7-10]. This investigation aimed to describe the echocardiographic characteristics of a cohort of centenarians at a tertiary referral center and to compare the findings between male and female patients.

## Materials and methods

Patient selection

The study protocol was drafted in accordance with institutional regulations and the ethical guidelines of the 1975 declaration of Helsinki and was approved by the Mount Sinai Medical Center Institutional Review Board in Miami Beach, Florida. We retrospectively analyzed the Echocardiography digital database and identified 100 consecutive patients referred for echocardiography between January 2009 and December 2020 who were aged 100 years or older. The database includes both inpatient and outpatient echocardiograms, of which each represented approximately 50% of the present cohort. The indications for echocardiography were dyspnea on exertion, cerebrovascular accident, arrhythmias, and/or heart failure surveillance. There were no exclusion criteria with the exception of age less than 100 years. A review of the institutional electronic health records was performed to assess patient demographics, clinical risk factors and co-morbidities, and laboratory values.

Echocardiography

All transthoracic echocardiograms were performed using a GE cardiovascular ultrasound system (General Electric Healthcare, Waukesha, WI, USA). The assessment of left ventricular (LV) systolic function, estimation of the ejection fraction, and assessment of geometry were performed in accordance with the American Society of Echocardiography chamber quantification guidelines [11]. In the parasternal long-axis view, the LV internal diameters were measured at end-systole and diastole, and the interventricular septum and posterior wall thickness at end-diastole. LV mass was calculated and indexed to body surface area. The relative wall thickness was calculated as (2 x LV posterior wall thickness/LV internal end-diastolic diameter). The LV mass and relative wall thickness were used to categorize the ventricular geometry as normal, concentric remodeling, concentric hypertrophy, or eccentric hypertrophy.

The assessment of LV diastology was performed according to the American Society of Echocardiography guideline recommendations [12]. The transmitral inflow E-wave and A-wave velocities were measured using pulsed wave Doppler aligned at the tips of the mitral valve leaflets in the apical four-chamber view. The E/A ratio expressed the ratio of peak blood flow occurring in early diastolic LV relaxation to blood flow from late diastolic atrial contraction. The medial and lateral mitral annular velocities were measured using tissue Doppler imaging and averaged. The E-wave velocity/average e’ velocity ratio was used as an estimation of the LV filling pressure.

Valvular heart disease was assessed in a multi-parametric manner according to the American Society of Echocardiography native valve regurgitation and stenosis guidelines [13,14]. The valve lesions were graded as none/trace, mild, moderate, or severe. The transaortic pressure gradients were estimated from the modified Bernoulli equation: 4 x (peak velocity)^2^. The right ventricular systolic pressure was estimated from the addition of the peak tricuspid regurgitant pressure gradient (using the modified Bernoulli equation) and the right atrial pressure. Finally, the left atrial size was expressed as the anteroposterior diameter measured in the parasternal long-axis view at end-systole, and the volume index was estimated by the biplane method of discs.

Statistical methods

The variables are expressed as the mean ± 1 standard deviation, median and interquartile range, or as absolute number and percentage. An independent t-test was used to compare continuous data stratified between males and females, while a Mann-Whitney U-test was utilized for non-parametric variables. A two-tailed p-value was calculated to test for statistical significance. The statistical analyses were conducted using Statistical Package for Social Sciences, version 21 (IBM Corp., Armonk, NY).

## Results

Demographics and clinical characteristics

One hundred consecutive patients with a mean age of 101.5 ± 1.7 years were identified, of which 78 (78%) were female. The mean body mass index, systolic blood pressure, and diastolic blood pressure measured 23.5 ± 4.8 kg/m^2^, 126 ± 24 mmHg, and 69 ± 13 mmHg, respectively. The most common co-morbidities were hypertension (77%), coronary artery disease (46%), and congestive heart failure (42%), with a small minority of patients having undergone surgical or percutaneous coronary revascularization (6%). When compared with female patients, males had a larger body surface area (1.71 ± 0.17 vs 1.54 ± 0.19 m^2^, p < 0.001) and a greater serum creatinine level (1.45 ± 0.6 vs 1.18 ± 0.57 mg/dL, p = 0.05) (Table 1).

**Table 1:** Patient demographics and clinical characteristics *Data available for 53 patients.

Variable	Total (N = 100)	Male (N = 22)	Female (N = 78)	p-Value
Age (years)	101.5 ± 1.7	101.4 ± 1.7	101.5 ± 1.7	0.74
Body surface area (m^2^)	1.58 ± 0.2	1.71 ± 0.17	1.54 ± 0.19	<0.001
Body mass index	23.5 ± 4.8	23.1 ± 3.4	23.6 ± 5.1	0.64
African-American ethnicity	6 (6%)	0	6 (7.7%)	0.33
Systolic blood pressure (mmHg)	126 ± 24	121 ± 23	128 ± 24	0.27
Diastolic blood pressure (mmHg)	69 ± 13	67 ± 12	70 ± 14	0.48
Pulse pressure (mmHg)	58 ± 19	54 ± 19	59 ± 19	0.3
Creatinine (mg/dL)	1.24 ± 0.59	1.45 ± 0.6	1.18 ± 0.57	0.05
NT-proB-type natriuretic peptide (pg/mL)^*^	5832 (2765-11,681)	5317 (1669-14,422)	5949 (2947-12,103)	0.58
Hypertension	77 (77%)	14 (63.6%)	63 (80.8%)	0.09
Diabetes mellitus	13 (13%)	2 (9.1%)	11 (14.1%)	0.73
Congestive heart failure	42 (42%)	11 (50%)	31 (39.7%)	0.39
Atrial fibrillation	33 (33%)	9 (40.9%)	24 (30.8%)	0.37
Coronary artery disease	46 (46%)	11 (50%)	35 (44.9%)	0.67
Peripheral arterial disease	9 (9%)	1 (4.5%)	8 (10.3%)	0.68
History of smoking	12 (12%)	2 (9.1%)	10 (12.8%)	1.0
Prior coronary revascularization	6 (6%)	1 (4.5%)	5 (6.5%)	1.0
Prior valvular surgery	3 (3%)	1 (4.5%)	2 (2.6%)	0.53

Left ventricular geometry, function, and diastology

The mean LV ejection fraction measured 56.9 ± 11.3%, and notably was greater in females when compared with males (58.4 ± 9.8% vs 51.6 ± 14.6%, p = 0.01). Male patients also had a higher prevalence of severe LV systolic dysfunction, defined as an ejection fraction <30% (18.2% vs 3.8%, p = 0.04); larger end-diastolic (2.8 ± 0.6 vs 2.5 ± 0.5, p = 0.03) and end-systolic diameter (1.9 ± 0.6 vs 1.6 ± 0.4, p = 0.001) indices; and a smaller relative wall thickness (0.54 ± 0.18 vs 0.69 ± 0.36, p = 0.06). The most common LV geometry patterns were concentric hypertrophy (46%) and remodeling (39%), with few patients exhibiting normal geometry (9%) or eccentric hypertrophy (6%); this was similar in both males and females. Finally, females had a higher E/e’ ratio consistent with a greater LV diastolic filling pressure, when compared with males (17.3 [13.2-23] vs 13.3 [10.3-19.6], p = 0.05) (Table 2).

**Table 2:** Left ventricular geometry, function, and diastology assessed by two-dimensional transthoracic echocardiography LV = left ventricle. Data available for *84 patients, **79 patients, and ^ll^87 patients, respectively.

Variable	Total (N = 100)	Male (N = 22)	Female (N = 78)	p-Value
Left ventricular size and function				
Left ventricular ejection fraction (%)	56.9 ± 11.3	51.6 ± 14.6	58.4 ± 9.8	0.01
Left ventricular ejection fraction >50%	85 (85%)	15 (68.2%)	70 (89.7%)	0.01
Left ventricular ejection fraction 41-50%	4 (4%)	3 (13.6%)	1 (1.3%)	0.03
Left ventricular ejection fraction 31-40%	4 (4%)	0	4 (5.1%)	0.57
Left ventricular ejection fraction <30%	7 (7%)	4 (18.2%)	3 (3.8%)	0.04
LV internal diastolic diameter index (cm/m^2^)	2.6 ± 0.5	2.8 ± 0.6	2.5 ± 0.5	0.03
LV internal systolic diameter index (cm/m^2^)	1.7 ± 0.5	1.9 ± 0.6	1.6 ± 0.4	0.001
Interventricular septal thickness (mm)	12.9 ± 3.8	12.0 ± 3.2	13.2 ± 3.9	0.21
Posterior wall thickness (mm)	12.1 ± 3.2	12.2 ± 3.3	12.1 ± 3.2	0.95
Left ventricular mass index (g/m^2^)	109.8 ± 42.7	121.6 ± 51.2	106.5 ± 39.8	0.15
Relative wall thickness	0.66 ± 0.34	0.54 ± 0.18	0.69 ± 0.36	0.06
Left ventricular geometry				
Normal	9 (9%)	4 (18.2%)	5 (6.4%)	0.1
Concentric remodeling	39 (39%)	8 (36.4%)	31 (39.7%)	0.77
Concentric hypertrophy	46 (46%)	9 (40.9%)	37 (47.4%)	0.59
Eccentric hypertrophy	6 (6%)	1 (4.5%)	5 (6.4%)	1.0
Diastology				
E-wave velocity (m/s)^*^	0.97 (0.75-1.28)	0.88 (0.68-0.97)	1.03 (0.76-1.28)	0.1
A-wave velocity (m/s)^**^	0.99 (0.64-1.25)	0.93 (0.69-1.11)	1.03 (0.6-1.31)	0.49
E/A-wave ratio	0.9 (0.7-1.7)	1.0 (0.7-1.4)	0.9 (0.7-1.9)	0.89
Average mitral annular velocity (m/s)^ll^	0.06 (0.05-0.07)	0.06 (0.06-0.07)	0.06 (0.05-0.07)	0.2
E/e' ratio^*^	16.2 (12.6-22)	13.3 (10.3-19.6)	17.3 (13.2-23)	0.05
Left atrial volume index (cm/m^2^)	34 ± 20	34 ± 17	34 ± 20	0.55
Left atrial diameter index (cm/m^2^)	2.6 ± 0.6	2.4 ± 0.8	2.6 ± 0.6	0.13
Right ventricular systolic pressure (mmHg)	44.9 ± 14.3	42.7 ± 12.6	45.5 ± 14.7	0.45

Valvular heart disease

The most common valvular heart disease was mitral and tricuspid regurgitation, which were present in 94% and 93% of patients. However, severe regurgitant lesions were noted in only a small minority, with a prevalence of 13.8% for mitral regurgitation and 12.9% for tricuspid regurgitation, respectively. Aortic stenosis was present in 33% of patients, with one-third of them having severe stenosis. While the prevalence of severe stenosis was similar between males and females, the overall estimated transaortic pressure gradients were significantly higher in female patients (mean gradient: 32.0 ± 17.7 vs 18.7 ± 9.2 mmHg, p = 0.04). The least common pathology was mitral stenosis (3%). The three patients who had undergone prior valve surgery had a bioprosthetic aortic valve replacement, and all prosthetic valves were functioning normally (Table 3).

**Table 3:** Valvular heart disease and lesion severity assessed by two-dimensional transthoracic echocardiography Prevalence of lesion severity is presented as a percentage of cases within each specific pathology.

Variable	Total (N = 100)	Male (N = 22)	Female (N = 78)	p-Value
Mitral annular calcification	78 (78%)	15 (68.2%)	63 (80.8%)	0.21
Mild	37 (47.4%)	10 (66.6%)	27 (42.9%)	
Moderate	24 (30.8%)	3 (20.0%)	21 (33.3%)	
Severe	17 (21.8%)	2 (13.3%)	15 (23.8%)	
Mitral stenosis	3 (3%)	0	3 (3.8%)	1.0
Mild	2 (66.7%)	0	2 (66.7%)	
Severe	1 (33.3%)	0	1 (33.3%)	
Mitral regurgitation	94 (94%)	21 (95.5%)	73 (93.6%)	0.75
Mild	64 (68.1%)	17 (81%)	47 (64.4%)	
Moderate	17 (18.1%)	1 (4.8%)	16 (21.9%)	
Severe	13 (13.8%)	3 (14.2%)	10 (13.7%)	
Aortic stenosis	33 (33%)	9 (40.9%)	24 (30.8%)	0.37
Mild	10 (30.3%)	3 (33.3%)	7 (29.2%)	
Moderate	11 (33.3%)	5 (55.6%)	6 (25%)	
Severe	12 (36.4%)	1 (11.1%)	11 (45.8%)	
Aortic regurgitation	59 (59%)	12 (54.5%)	47 (60.3%)	0.63
Mild	49 (83.1%)	11 (91.7%)	38 (80.9%)	
Moderate	10 (16.9%)	1 (8.3%)	9 (19.1%)	
Tricuspid regurgitation	93 (93%)	20 (90.9%)	73 (93.6%)	0.66
Mild	67 (72%)	15 (75.0%)	52 (71.2%)	
Moderate	14 (15.1%)	4 (20.0%)	10 (13.7%)	
Severe	12 (12.9%)	1 (5.0%)	11 (15.1%)	

## Discussion

In the present cross-sectional echocardiographic study of centenarians, the following findings were noted: 1) the majority of patients were female, with the overall cohort exhibiting a healthy body mass index and blood pressure; 2) as a group, 85% of centenarians had a preserved LV size and systolic function, and the vast majority either concentrically remodeled or hypertrophied ventricles; 3) as a group, there was a mild elevation in the mean LV filling pressure and mild pulmonary hypertension, as assessed by the E/e’ ratio and right ventricular systolic pressure; 4) mild valvular heart disease was common, with mitral and tricuspid regurgitation being the most prevalent lesions observed; and 5) females had a greater LV ejection fraction, a smaller chamber size, a higher filling pressure, and greater transaortic stenotic pressure gradients when compared with males.

Given their longer documented life expectancy it is not surprising that females represented the majority of centenarians in the present study; indeed, the female prevalence in prior small centenarian cohorts has ranged from 60% to 85% [7-9]. Significant differences existed in LV size, systolic function, and estimates of filling pressure between sexes. Females exhibited smaller chambers with a greater ejection fraction and higher E/e’ ratio, consistent with patterns observed in the decades of aging preceding centenarians [15-17]. In the PIVUS study (Prospective Investigation of the Vasculature in Uppsala Senior), investigators enrolled 922 patients aged 70 years, of which 462 (50.1%) were female, with all participants undergoing comprehensive two-dimensional (2D) echocardiography [16]. As in the present cohort, females had a greater LV ejection fraction (68% vs 65%, p = 0.0008), a smaller end-diastolic diameter (45 vs 49 mm, p < 0.001), and more abnormal diastology. Despite these differences, the extent of LV remodeling and geometry patterns were similar between the sexes in both the PIVUS study and the present centenarian cohort. However, in the PIVUS septuagenarians, a normal LV geometry was present in approximately 50% of individuals, with the concentric remodeling in 25% and concentric LV hypertrophy in 18%. The cohort of centenarians exhibited far greater concentric hypertrophy at 46% and concentric remodeling at 39%, at the expense of a normal LV geometry pattern. Placed within this context, it can be hypothesized that gross measures of LV size and function may differ in males and females due to inherited and acquired factors, with the net result of LV remodeling occurring in a similar adaptive and progressive fashion during aging.

The most prevalent cardiovascular co-morbidity in the present cohort was hypertension, which was observed in 77% of patients, and is similar to prior registry data [7-10]. Aging is associated with vascular changes at the level of the arterial wall media as well as the endothelium, which reduces arterial distensibility and promotes increased vascular stiffness [18]. For this reason, systolic blood pressure tends to rise with age, even in normotensive populations. The mean blood pressure in the cohort at the time of echocardiography was in the normal range, likely reflecting a combination of appropriate treatment and delayed onset or mild age-related hypertension. Furthermore, as noted, the pattern of LV remodeling observed was concentric remodeling or hypertrophy in 85%, which has been shown to be an earlier or compensatory adaptation to hemodynamic stressors and imparts a better prognosis than eccentric hypertrophy or remodeling [19,20].

Congestive heart failure is one of the leading causes of death in the centenarian population, with the majority of patients having a preserved LV ejection fraction (HFpEF) [7,8,10]. As a preserved LVEF >50% was present in 85% of the cohort patients, it can be inferred that HFpEF was the clinical paradigm most prevalent among the 42% who had a history of congestive heart failure in the present study. HFpEF almost invariably presents with abnormalities in diastolic function, which includes impaired LV relaxation, myocardial stiffening and reduced compliance, increased LV filling pressure, and left atrial remodeling [21]. This manifested in the present centenarians as a mean E/e’ ratio of 16.2, right ventricular systolic pressure of 45 mmHg, and left atrial volume index of 34 mL/m^2^, and a median NT-proB-type natriuretic peptide of 5832 pg/mL. Collectively these findings are indicative of mildly elevated LV filling pressures, mild pulmonary hypertension, and LV myocardial wall stress. Of note, females were found to have a more elevated LV filling pressure when compared with males, which has been noted to differentiate the HFpEF phenotype between genders [22].

The prevalence of valvular heart disease in older adults has risen exponentially over the last several decades owing to an aging population in an era of improved medical and interventional therapies. Approximately 10% to 15% of patients aged 75 years or greater have at least one moderate to severe valvular lesion, and it is well established that morbidity and mortality are commensurate to regurgitant or stenotic severity [23]. Similarly, and despite being substantially older, the centenarians had a prevalence of 13.5% for severe valvular heart disease. The most common severe lesions were aortic stenosis, mitral regurgitation, and tricuspid regurgitation; only three patients had required surgical valve intervention for symptomatic aortic stenosis. Interestingly, the average mean transaortic pressure gradient was significantly higher in females when compared with males (32 vs 19 mmHg, p = 0.04). While LV remodeling as stratified by geometric adaptation was similar between genders, females were noted to have a greater LVEF, smaller chambers, and a greater relative wall thickness, and have been shown in prior studies to have stenotic valves characterized by greater fibrosis as opposed to the marked calcific changes observed in men [24]. As such, it is hypothesized that the female LV may adapt more favorably to aortic stenosis-related pressure overload and thus generates greater transaortic pressure gradients compared with males.

There are limitations to the present study that should be considered when interpreting the findings. First, the cross-sectional nature of the study provides an analysis of variables only at a single point in time. This does not allow for a temporal outcomes analysis, and findings cannot be used to imply causality. Secondly, given the population studied, the sample size was relatively small. This limits the power to identify differences between genders in the statistical analyses, which is also exacerbated by the imbalance between the number of male versus female patients. Thirdly, data regarding the medical management of co-morbidities were not available, which may impact measured hemodynamic, clinical, and echocardiographic variables. Fourthly, not all patients had data available for analysis with regard to diastology and serum levels of NT-proB-type natriuretic peptide. This is a form of selection or attrition bias and represents an uncontrollable confounder. Fifthly, while valvular lesions were assessed and graded in a multi-parametric manner, detailed quantitative parameters were not independently captured in the current study with the exception of the transaortic pressure gradients among patients with aortic stenosis. While a multi-parametric approach allows for the integration of all available qualitative and hemodynamic measures of valvular function, it should be noted that interobserver variability cannot be excluded.

## Conclusions

In conclusion, the present study on centenarians affords a cross-sectional clinical and echocardiographic evaluation of a growing and under-studied population within cardiovascular medicine and highlights important differences between male and female cardiac structure and function. Future prospective and registry studies of centenarians will be important in identifying the natural history of their co-morbidities, as well as the management of their heart failure, diastolic dysfunction, and valvular heart disease.
